# Immune thrombocytopenia management during COVID‐19 pandemic: An Italian monocentric experience

**DOI:** 10.1002/jha2.406

**Published:** 2022-03-09

**Authors:** Gianfranco Lapietra, Antonietta Ferretti, Erminia Baldacci, Antonio Chistolini, Cristina Santoro

**Affiliations:** ^1^ Division of Hematology Department of Translational and Precision Medicine Policlinico Umberto I, Sapienza University of Rome Rome Italy

**Keywords:** COVID‐19, ITP, management, SARS‐CoV2, therapy

## Abstract

Over the last 2 years, different cases of immune thrombocytopenia (ITP) in patients affected by SARS‐CoV2 have been reported. The management of SARS‐CoV2 in subjects with simultaneous or previous ITP can be challenging because of the great involvement of the haemostatic system in this viral infection. In this report, we describe the management and outcome of patients with newly diagnosed (ND), chronic and previous ITP, infected by COVID‐19, referred to the Haematology Institute of University Hospital Policlinico Umberto I in Rome. Steroids + immunoglobulins for ND or relapsed ITP and continuation of home therapy for chronic ITP are advised, although further knowledge is required.

1

Immune thrombocytopenia (ITP) is an acquired immune disorder characterised by a platelet count of less than 100 × 10^9^/L, leading to an increased bleeding risk. It can occur both in children and adults with an incidence of two to five per 100,000 persons in the general population, with a slight female preponderance [1]. The pathogenesis of ITP is not fully understood yet, but it seems to be triggered by environmental factors in subjects with inherited predisposition. All infectious diseases may potentially cause ITP but the main association has been observed with viral agents [1]. Since the start of the novel pandemic caused by severe acute respiratory syndrome coronavirus‐2 (SARS‐CoV2), known as COVID‐19, different cases of ITP in infected subjects have been reported from all over the world. Most of these reports are describing newly diagnosed ITP (ND‐ITP) in COVID‐19 patients [2, 3, 4, 5, 6, 7]; some authors also report subjects with chronic ITP infected with SARS‐CoV2, either relapsing or not [8, 9].

During the first phase of pandemic (February–June 2020), in our Haematology centre in Rome we did not observe any case of concomitant ITP and COVID‐19 from outpatient clinic. This could reflect the predominantly nosocomial distribution of the infection at the beginning of the emergency in Italy. During the second phase, 17 ITP patients, in different phases of the disease, were infected by SARS‐CoV2. All these subjects had RT‐PCR confirmed SARS‐CoV2 infection on a nasopharyngeal swab performed between October 2020 and January 2021. Six were males (35.3%) and 11 females (64.7%). The median age was 57 years (range 30–90). At the time of the COVID‐19 infection, patients were characterised as follows: three had simultaneous ND‐ITP (17.6%) and one chronic patient experienced a first relapse (5.8%) (median platelet count 5.5 × 10^9^/L, range 2–30); seven subjects had chronic ITP on treatment (41.2%) (eltrombopag, *n* = 5; romiplostim, *n* = 1; prednisone, *n* = 1) (median platelet count 48 × 10^9^/L, range 31–99); two cases had stable chronic ITP never treated (11.8%) (median platelet count 72 × 10^9^/L, range 55–90); four subjects had chronic ITP off therapy on follow‐up (FU) (23.6%) (platelet count >100 × 10^9^/L; median FU 92.5 months, range 12–167). Twelve of 14 (70.5%) chronic ITP patients previously received one to three therapy lines (only steroids, *n* = 4; steroids + intra‐venous immunoglobulins [IvIg], *n* = 4; steroids + splenectomy, *n* = 2; steroids + IvIg + splenectomy, *n* = 2) (Table 1).

**TABLE 1:** Patients characteristics

Number of patients	17
Sex	Six males (35.3%); 11 females (64.7%)
Median age (years)(range)	57 (30–90)
Phase of ITP	Three ND‐ITP (17.6%)
	One relapse in chronic ITP (5.8%)
	Seven chronic ITP on treatment with oral steroids (1) or with TPO‐RAS (6) (41.2%)
	Two stable chronic ITP never treated (11.8%)
	Four chronic ITP off therapy (23.6%)
No. of previous therapy lines for ITP	One: corticosteroids → 4 patients (23.6%)
	Two: corticosteroids + immunoglobulins → 4 patients (23.6%)
	Two: corticosteroids + splenectomy → 2 patients (11.8%)
	Three: corticosteroids + immunoglobulins+splenectomy → 2 patients (11.8%)
Therapy for SARS‐CoV‐2	One: corticosteroids + antibiotics (macrolides) → 6 patients (35.3%)
	Two: antipyretics (paracetamol) → 11 patients (64.7%)

Abbreviations: ND‐ITP, newly diagnosed ITP;

TPO‐RAs, thrombopoietin receptor agonists.

Overall, 15 subjects presented either COVID‐19 infection‐related symptoms (fever, anosmia, dysgeusia, articular pain and mild‐to‐moderate respiratory distress) or bleedings (bruising, petechiae and epistaxis). In particular, 11 of them had only COVID‐related symptoms (64.8%), one had isolated mucocutaneous bleeding (5.8%) and three reported both (17.6%). Only two did not report any symptoms throughout the course of the infection (11.8%) (Figure 1).

**FIGURE 1:**
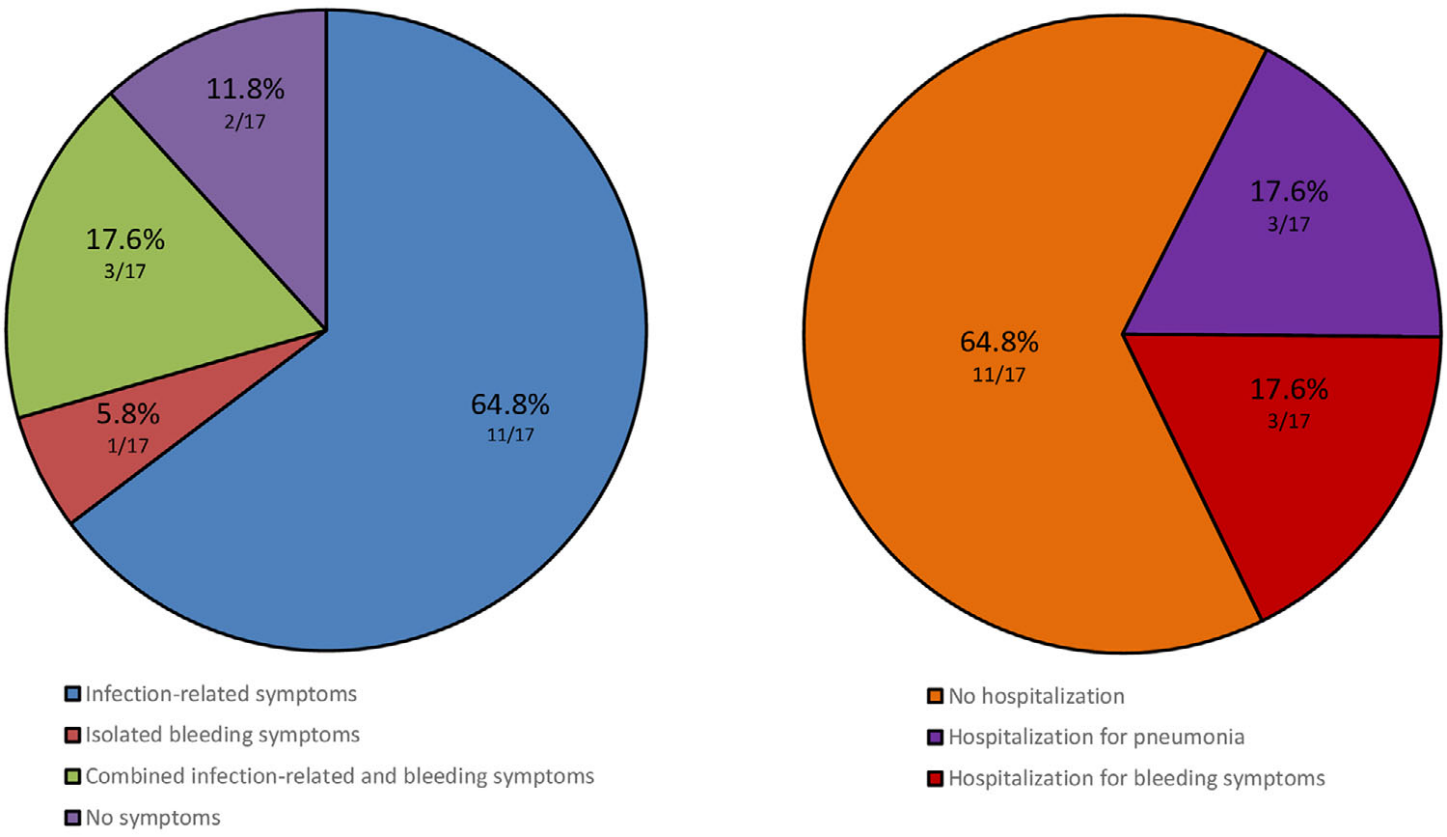
Clinical presentation and hospitalization rate in COVID‐19 and immune thrombocytopenia (ITP)

Six patients required hospitalization (35.2%): three for rapid decrease of platelet count and mucocutaneous bleeding (17.6%) (ND‐ITP = 2; ITP relapse = 1) and the other three for pneumonia (17.6%) (ND‐ITP = 1; chronic ITP on treatment with low dose prednisone, after induction with high dose in 2012 = 1; chronic ITP off treatment [previously exposed to the high dose of steroid in 2009 and 2012, then splenectomised in 2013] on FU = 1). The three subjects requiring hospitalization for ITP and related bleeding symptoms obtained a response to the following treatment: IvIg (1 g/kg, day 1) and steroids (dexamethasone 40 mg/day, days 1–4, followed by prednisone 0.5–1 mg/kg/day tapered after 15 days). Among patients hospitalised for pneumonia, the one who developed ND‐ITP responded to oral prednisone 1 mg/kg/day for 2 weeks and then tapered. The other two received antibiotics and corticosteroids just for the treatment of pneumonia as indicated by the treatment infectious protocols adopted. The median duration of hospitalization was 10.5 days (range 3–20). After hospital discharge, steroids were gradually reduced, based on clinical and laboratory evaluations. In particular, we performed a weekly follow‐up in the first month, then every 2 weeks, finally every month and, to date, no complications have been observed. Eleven patients recovered at home without any bleeding (64.8%) (Figure 1). During quarantine, none of them could check platelet count. Soon after, at the first evaluation, only two subjects presented with asymptomatic reduction in platelet count, compared to their baseline condition: In the first case, the platelets dropped from 89 × 10^9^/L to 22 × 10^9^/L, and in the other case, it dropped from 249 × 10^9^/L to 79 × 10^9^/L. These variations were successfully managed with little adjustment of thrombopoietin‐receptor agonists (TPO‐RAs) dosage. No cases of reactive thrombocytosis were observed.

Platelet‐count adjusted prophylaxis of COVID‐19‐related thromboembolism was not used in non‐hospitalised patients due to clinical decision; among the six hospitalised cases, two with chronic ITP had platelet count >50 × 10^9^/L (161 and 103 × 10^9^/L, respectively): one was on anticoagulant therapy for atrial fibrillation, and the other one was on antiplatelet treatment for metabolic syndrome. These therapies were maintained during hospitalization. The other four hospitalised patients did not receive any anti‐thrombotic prophylaxis because of a very low platelet count. Overall, no cases of either symptomatic thrombosis or serious respiratory distress were observed. The outcome was favourable in all the patients and no death was reported. The viral seroconversion, defined as detectable anti‐spike IgG in the blood at the first control after negative nasopharyngeal swab, occurred in all subjects. To date, the whole cohort received at least two doses of a specific available RNA vaccine without complications or variations in the platelet count.

In spite of the small sample size, we would like to highlight some aspects. First, the two cases of ND‐ITP and the single case of relapse requiring hospitalization were responsive to IvIg and steroids therapy. This relies on the immune nature of this kind of thrombocytopenia in our patients affected by COVID‐19. Xu et al. described different causes that might explain the frequent association between the new respiratory infection and a low platelet count [10]. SARS‐CoV2 could either increase the platelet consumption by activating the coagulation cascade subsequent to lung endothelial damage or invalidate thrombocytopoiesis by infiltrating the bone marrow niche. On the other side, pure ITP seems to be due to platelet destruction for the cross‐reaction between the anti‐COVID‐19 IgG and the platelet membrane components. If this immune dysregulation occurs, the response to corticosteroids and Ig could be favourable, like in our experience and in other cases reported in literature [2, 3, 4, 5, 6, 7]. The practical guidance for the management of adult ITP during the COVID‐19 pandemic also endorses this evidence. In particular, in patients with new or relapsed acute ITP, corticosteroids should be started at a dose of 20–25 mg daily in non‐bleeding subjects, with an increase of 1 mg/kg (up to a maximum of 80 mg/day) after 3–5 days in absence of response. Prolonged administrations should be avoided, and tapering should be initiated within 2 weeks after the start of therapy. Intravenous Ig (1 g/kg) may be necessary if immediate elevation of the platelet count is required to control bleeding [11, 12]. In our patients with ITP‐related bleeding symptoms, IvIg and a higher than the recommended dose of steroids were started to obtain a quick response on platelet count. The outcome was anyway positive.

Another interesting observation regards the outcome of COVID‐19 infection in 12 chronic patients with stable on treatment or off‐therapy ITP. Among them, six were on TPO‐RAs, one was on low doses of prednisone and five were off treatment since a median time of 92.5 months (range 12–167): only two of 12 (16.6%) required hospitalization for pneumonia. The off‐therapy subjects with the previous exposition to different therapy lines, including steroids and splenectomy, did not present with a worse outcome. Anyhow, we have to highlight that splenectomies were all performed >5 years since the SARS‐CoV2 infection and that none of the patients had previously been treated with rituximab. The impact of splenectomy on COVID‐19 is controversial, since this surgery procedure seems to increase more the risk of bacterial than viral infections; after rituximab, severe infections caused by hepatitis B virus, cytomegalovirus, Herpes zoster and *Pneumocystis jirovecii* have been reported and some clinicians prescribe prophylactic anti‐microbial therapy also in ITP cases [13]. Regarding patients on therapy, the low rate of infection in those treated with TPO‐RAs is consistent with data from literature showing relative safety of this class of drugs [14, 15]. The no need for discontinuation of chronic treatment with TPO‐RAs during SARS‐CoV2 infection is stated by both national and British guidelines even if the increased thromboembolic and transaminitis risk should be considered and close monitoring of patients should be done [11, 12].

Early thrombocytosis after SARS‐CoV2 infection in subjects with chronic ITP on treatment with need for therapy reduction or even discontinuation has been reported in other case series [10]. In our experience, no patients presented with a paradoxical increase of platelets and no symptomatic thrombosis occurred despite the absence of a specific prophylactic therapy. In fact, antiplatelet or anticoagulant prophylaxis was avoided either in hospitalized patients or in homecare ones, except in two already on such therapies. This decision was made also in consideration of the absence of thrombotic risk factors beyond the viral infection. Symptomatic thrombotic complications did not occur even in subjects on treatment with TPO‐RAs again confirming the relative safety of these biologic drugs.

In conclusion, we would like to highlight the potential implications of our data, which provides an interesting perspective within the current pandemic. If COVID‐19 infection is associated with new‐onset of thrombocytopenia, ITP must be taken into consideration in the differential diagnosis, especially if the platelet reduction is isolated, without any variation in the coagulation parameters. On the other hand, in case of new onset of ITP, it could be recommended to search for COVID‐19 infection. Corticosteroids and IvIg can be successfully used also in these cases. The management of COVID‐19 infection in patients with a known ITP does not differ from the normal population and there is no need for discontinuation of the chronic treatment.

Further knowledge would be useful to improve the management of concomitant ITP and COVID‐19 infection.

## CONFLICT OF INTEREST

The authors declare no conflicts of interest.

## AUTHOR CONTRIBUTIONS

All the authors equally contributed to the final version of the manuscript and approved it.
